# Supplementary material for: Tau seeding and spreading in vivo is supported by both AD-derived fibrillar and oligomeric tau
Source: Acta Neuropathol. 2023 Jun 21;146(2):191–210. doi: 10.1007/s00401-023-02600-1 (PMC10329061; doi:10.1007/s00401-023-02600-1)
Supplement: Supplementary file 1 — Supplementary file1 (PDF 17 kb) [file 401_2023_2600_MOESM1_ESM.pdf]

Antibody	Reactivity	Species	Reference	IHC dilution	IF dilution	WB dilution
**AT8 (biotinylated)**	Human Tau pS212, pT214	Mouse	Thermofisher (MN1020B)	1:400	1:400	
**AT100**	Human Tau pS202, pT205	Mouse	Thermofisher (MN1060)	1:4000	1:1000	
**Bassoon**	Mouse presynaptic	Guinea pig	Synaptic systems (141 004)		1:1500	
**Clec7a (murine)**	murine Dectin-1	Rat	InvivoGen		1:50	
**GFAP-CY3**	GFAP	Mouse	Sigma-Aldrich (C9205)		1:10,000	
**Iba1**	Iba1	Rabbit	Wako (019-19741)		1:1000	
**Iba1**	Iba1	Rabbit	Abcam (ab178847)	1:500 (autostainer)		
**pS422**	Human Tau pS422	Rabbit	Abcam (ab79415)		1:1000	
**PSD95**	Mouse postsynaptic	Rabbit	Thermofisher (51-6900)		1:1000	
**Tau DAKO**	Total Tau	Rabbit	DAKO (A0024)			1:5000

Table S1: list of the primary antibodies used in the present study and their working concentration.
